# What happens to the lower lumbar spine after marathon running: a 3.0 T MRI study of 21 first-time marathoners

**DOI:** 10.1007/s00256-021-03906-5

**Published:** 2021-09-20

**Authors:** Laura M. Horga, Johann Henckel, Anastasia Fotiadou, Anna Di Laura, Anna C. Hirschmann, Robert Lee, Alister J. Hart

**Affiliations:** 1 Institute of Orthopaedics and Musculoskeletal Science, University College London and the Royal National Orthopaedic Hospital, Stanmore, London, UK; 2 Department of Radiology and Nuclear Medicine, University Hospital Basel, Basel, Switzerland; 3 Department of Spinal Surgery, Royal National Orthopaedic Hospital, Stanmore, London, UK

**Keywords:** Running, Marathon, Spine, Pelvis, MRI

## Abstract

**Objective:**

To better understand the impact of long-distance running on runners’ lumbar spines by assessing changes before and after their first marathon run.

**Materials and methods:**

The lumbar spines of 28 asymptomatic adults (14 males, 14 females, mean age: 30 years old), who registered for their first marathon, the 2019 London Richmond Marathon, were examined 16 weeks before (time point 1) and 2 weeks after (time point 2) the marathon. Participants undertook a pre-race 16-week training programme. Magnetic resonance imaging (MRI) of high-resolution 3.0 Tesla was used at each time point. Senior musculoskeletal radiologists assessed the lower lumbar spine condition.

**Results:**

Out of 28 participants, 21 completed both the training and the race and 7 neither completed the training nor started the marathon but not due to spine-related issues. At time point 1, disc degeneration was detected in 17/28 (61%), most predominantly at spinal segments L4–L5 and L5–S1. No back pain/other symptoms were reported. When compared to time point 2, there was no progression in the extent of disc degeneration, including intervertebral disc (IVD) height (*p* = 0.234), width (*p* = 0.359), and intervertebral distance (*p* = 0.641). There was a regression in 2 out of 8 (25%) participants who had pre-marathon sacroiliac joint bone marrow oedema, and a small increase in the size of a pre-marathon subchondral cyst in one participant, all asymptomatic.

**Conclusion:**

Running 500 miles over 4 months plus a marathon for the first time had no adverse effects on the lumbar spine, even when early degenerative changes were present. Additionally, there was evidence of regression of sacroiliac joint abnormalities.

**Supplementary Information:**

The online version contains supplementary material available at 10.1007/s00256-021-03906-5.

## Introduction

Long-distance running is extremely popular, with over a million runners participating in marathon runs (42 km) each year [1]. Despite the well-known cardiorespiratory benefits of running, there have been concerns related to the impact of marathon running on lumbar spine health, especially on intervertebral disc (IVD) conditions. During running, significant compression and rotational forces are exerted on the lower lumbar discs; however, it is yet unclear whether repetitive running as in a marathon is harmful to the lumbar spine [2, 3].

Magnetic resonance imaging (MRI) is an important tool in identifying, grading, and categorising the level of disc degeneration (Pfirrmann grading system) [4–6]. Moreover, the MRI scans can be effectively used to measure lumbar spine features, including morphological alterations to the IVD such as height and width [4]. In particular, high-resolution 3.0 Tesla (T) MRI provides unprecedented accuracy in assessing lumbar spine conditions [7, 8].

The literature on MRI-based research of the impact of running on lumbar spine health is very scarce and no study has investigated marathon running. There are conflicting results with firstly, positives outcomes in intervertebral disc health [9, 10] in middle-aged endurance runners with a long history of running and high weekly mileage [3]. And secondly, negative outcomes in moderate-intensity running resulting in intervertebral disc compression in young adults [11] and spinal disc degenerative changes in Olympic athletes [12]. However, the study on Olympic athletes shows the importance of sequential MRI scanning and recruitment of asymptomatic participants, as many had a history of previous injuries which could have made them more vulnerable to degenerative changes [12].

The objective of our study was to evaluate the 3.0 T MRI lumbar spine findings of first-time marathon runners before and after a 4-month training programme ending in a marathon race and thus to better understand whether this running dose is harmful to lumbar spine health.

## Methods

### Participants and methods

This was a prospective, longitudinal cohort study with individuals who signed up for the Richmond Marathon 2019. The research study was approved by a Research Ethics Committee (REC) 13,823/001. All participants gave written informed consent before taking part in the study.

Inclusion criteria included: no present or previous lumbar spine injuries or surgeries; no symptoms related to their musculoskeletal condition; no previous marathon runs; no contraindications to MRI. The main exclusion criteria were: pregnancy, active breastfeeding, age < 18 years old, claustrophobia, and history of panic attacks or anxiety, known lumbar spine problems.

Twenty-eight volunteers who registered to run their first marathon ever, the Richmond Marathon 2019, were recruited to the study (14 males, 14 females; median age: 30 years, range: 18–58 years old). Basic demographics were collected at baseline: weight (70.4 ± 9.6 kg), height (174 ± 10.2 cm), and body mass index (BMI). All participants reported similar previous running experiences: they previously participated in races ranging from 10 km up to half-marathon (21 km) distances, and never ran a marathon (42 km) before. Specifically, 5 people ran a 10 km race as their longest distance and 23/28 ran a half-marathon as their longest distance race, running ≥ 2 times/week (median: 3; range: 2–5 times/week), for a total of 3–4 h of running/week.

All participants started a formal 4-month training programme for the marathon provided by the race organiser (with a gradual increase in mileage/week, available online on the Richmond Marathon website). All underwent lower lumbar spine MRI scans prior to the start of the training plan (time point 1).

A number of 21/28 participants completed both the training for the marathon and the marathon run itself. Following the marathon run, participants were invited to attend a second MRI scan (time point 2).

### MRI protocol

The participants had lower lumbar spine 3.0 T MRI scans (Siemens Healthineers-Magnetom Vida, Erlangen, Germany) before and after running a marathon with a dedicated 18 channel ultraflex coil. The spine section being captured by MRI scanning was L3–S1 (comprising of lumbar vertebrae L3, L4, L5, and sacral vertebra S1). The MRI protocol included the following sequences: fat-suppressed proton-density-weighted turbo spin-echo (FS PDw TSE) sequences in coronal [repetition time (TR): 4190 ms/echo time (TE): 44 ms; image size/acquisition matrix: 512 × 512 pixels; field of view (FOV): 70.8 × 30 cm] and sagittal bilateral planes proton density [FS TSE TR: 4420/TE: 35 (320 × 320 pixels); FOV: 82.6 × 35 cm]– ‘bilateral’ implies that scanning on sagittal slices was performed from right to left on a single acquisition; axial (T1 TSE TR: 27/TE: 10; FOV: 82.6 × 35 cm) covering the lower lumbar spine; coronal PD TSE (TR: 3290/TE: 39; FOV: 69.8 × 29.6 cm); axial PD FS TSE [TR: 4400/TE: 36 (384 × 384 pixels); FOV: 82.6 × 35 cm] and axial Dixon in 4 phases (in-phase, out-of-phase, water only, and fat only; TR: 4220/TE: 45; FOV: 70.8 × 30 cm); T1 VIBE 3D coronal (TR: 0.1/TE: 4.92; FOV: 70.8 × 30 cm). The thickness of all non-Dixon slices was 3 mm, whilst the thickness of Dixon slices was 1.5 mm. The interslice gap used in sequences was 0.3 mm. The scanning time per individual was 30 min.

### Imaging analysis

The MRI scans were evaluated using a picture archiving and communications system (PACS) workstation by 2 senior musculoskeletal radiologists with 10-year experience at consultant level, both at time point 1 and time point 2: one radiologist reported the full set of scans and the second one co-reported images from 20% of the study participants (*n* = 6 participants × 2-time points), independently. Double-reporting was done to verify the reproducibility of the readings. The participants whose scans were double-reported were randomly selected. The % for co-reporting was internally decided; also in previous studies the scans of 10% of the total number of subjects were co-reported^6^, but in this study, the subset was doubled to 20% for increased reliability.

Time point 1 MRI scans were examined at that specific time point by each radiologist, separately. Then, at time point 2, both MRI scans of each participant were compared for changes between timepoint 1 and time point 2 by each individual radiologist, again independently. The order was known, yet the examinations were pseudonymised and systematically analysed. Radiologists were blinded to any of the participants’ clinical information.

If there were any differences between the 2 radiologists’ reports, a second MRI reporting session determined the final scores based on a consensus reading.

MRI findings of the lumbar spine were assessed based on validated scoring systems and specific measurements. The following lumbar spine features were assessed: intervertebral disc height (IVD height), intervertebral disc width (IVD width), intervertebral distance, and disc degeneration. The presence of other findings, such as insufficiency fracture, facet joint effusion, or other sacroiliac joint findings was specified.

Measurements of IVD dimensions were done based on Kingsley et al. [11]. The margins of the vertebral bodies were digitised for all MRI slices where the vertebral endplate and IVD could be detected. The points were interpolated, and the resulting coordinates were used to measure the distances between adjacent vertebral endplates were calculated and thus calculate mean vertical IVD height and width.

We assessed the frequency of disc disease degeneration of the lumbar spine, including different levels of severity using Pfirrmann’s classification[4] as in Table 1 below:

**Table 1:** Pfirrmann’s classification of disc degeneration

Grade	Description of disc condition
I	Homogeneous disc and bright hyperintense white signal; Clear distinction between nucleus and anulus; Normal disc height.
II	Inhomogeneous disc with or without horizontal bands but keeping the hyperintense white signal isointense to cerebrospinal fluid; Clear distinction between nucleus and anulus; Normal disc height.
III	Inhomogeneous disc with intermediate grey signal intensity; Unclear distinction between nucleus and anulus; Normal to slightly decreased disc height.
IV	Inhomogeneous disc with intermediate to hypointense grey to black signal intensity; No longer distinction between nucleus and anulus; Slightly or moderately decreased disc height.
V	Inhomogeneous disc with hypointense black signal intensity; No distinction between nucleus and anulus; Disc space is collapsed.

### Statistical analysis

Demographics and characteristics of study participants were evaluated, including gender, age, and BMI. Changes between time point 1 and time point 2 MRI-reported datasets were assessed using paired *t*-test. Distinctions were made in terms of lumbar spine outcomes between male and female participants, those aged < 40 years old and ≥ 40 years old, and those with BMI < 25 kg/m^2^ or BMI ≥ 25 kg/m^2^, respectively, using unpaired *t*-tests.

Differences between marathon finishers and training non-finishers were analysed using unpaired *t*-test. Marathon finishing times of participants with and without disc degeneration were compared with unpaired *t*-test.

Interreader agreement (between the scores reported by radiologists) was calculated based on kappa statistics. The interpretation of kappa values was the following: kappa < 0, less than chance agreement; 0.010–0.200, slight agreement; 0.210–0.400, fair agreement; 0.410–0.600, moderate agreement; 0.610–0.800, substantial agreement; 0.810–0.990, almost perfect agreement; 1.000, perfect agreement. Statistical significance was defined as *p* < 0.05 (GraphPad Prism, V.6.0 c).

## Results

### Time point 1 findings

**Fig. 1:**
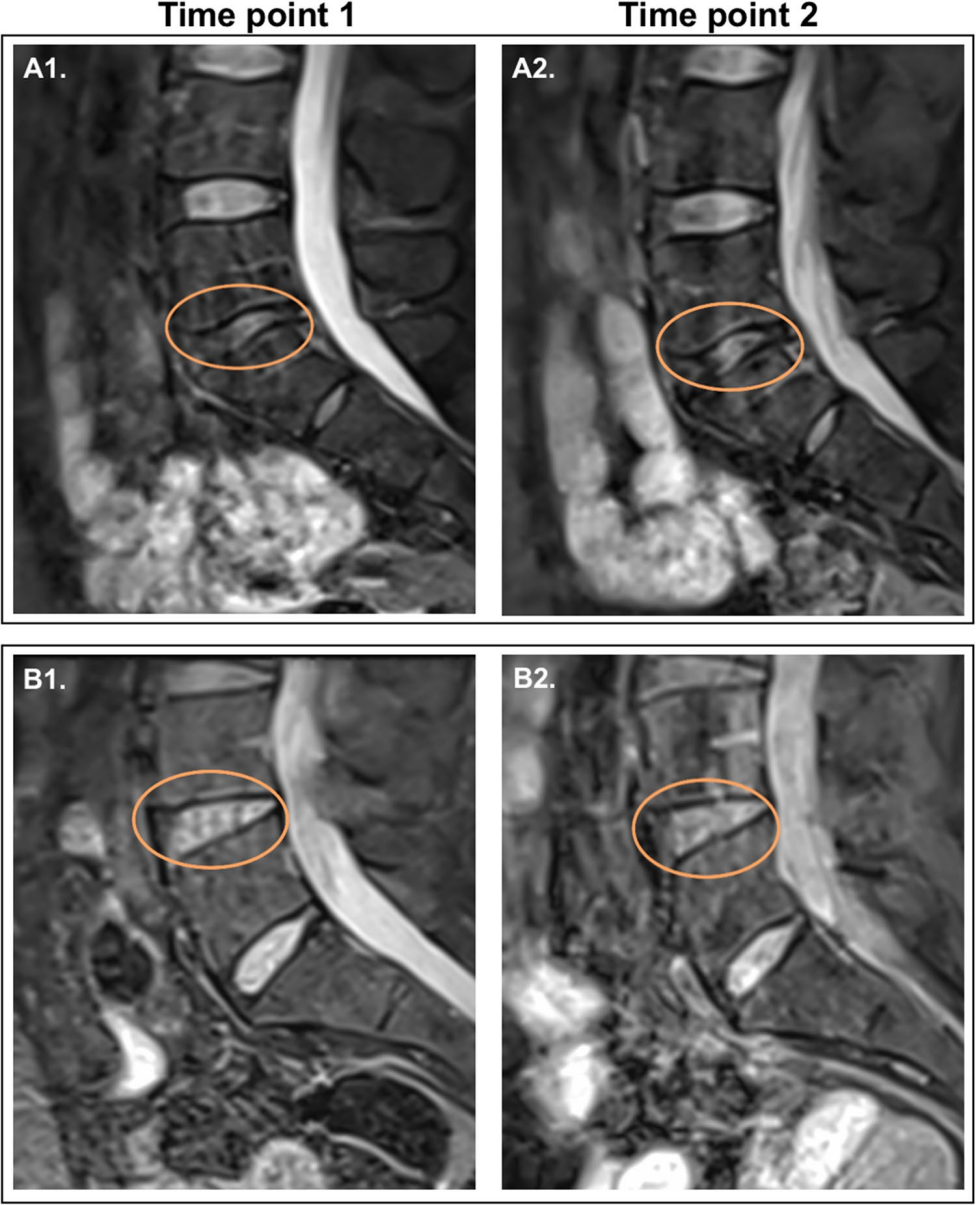
Sagittal PD FS TSE MR images of 2 asymptomatic participants: **A** moderate disc degeneration (grade 3) at L5–S1 at time point 1 (A1) and no worsening at time point 2 (A2) in a 45-year-old man; **B** mild disc degeneration (grade 2) at L4–L5 at time point 1 (B1) and no worsening at time point 2 (B2) in a 58-year-old woman

Before starting the training for the marathon, all 28 participants underwent a lumbar spine MRI scan. The MRI scans showed that disc degeneration (Pfirrmann grade > 1) was common amongst asymptomatic participants with no back pain: (17/28 61%). Mild degeneration (grade 2) was most prevalent (12/17; 71%), few participants had moderate disc degeneration (grade 3) (4/28; 14%), and one had severe degeneration (grade 4); see Table 2, Fig. 1.

**Table 2:** Participants with disc changes on MRI at time point 1 (*n* = 28 total cohort)

Disc	Discs with minimal changes (grade 1)	Discs with mild degeneration (grade 2)	Discs with moderate degeneration (grade 3)	Discs with severe degeneration (grade 4)	Discs with severe degeneration (grade 5)	Discs with degeneration, *n* (%)
L3–L4*	6	6	2	0	0	8 (42%)**
L4–L5	9	13	2	0	0	15 (54%)**
L5–S1	8	11	4	1	0	16 (57%)**
Any^β^	6	12	4	1	0	17 (61%)

*there were 9 cases where L3–L4 was not captured on scans, so the total number of assessed L3–L4 discs of participants was 19 instead of 28; **Percentages do not add up to 100% because participants may have more than one disc with degenerative appearances. ^β^Participants with disc changes in any of the L3–S1 vertebrae – highest grade was counted. *L*, lumbar vertebra; *S*, sacral vertebra.

The region most affected by disc degeneration was L4–S1: 15/28 participants had such appearances in both L4–L5 and L5–S1 simultaneously (54%).

### Training for and marathon completion

Out of 28 participants, 21 completed both the marathon training and the race itself (marathon finishers) whilst 7 interrupted their training and did not enter the race (training non-finishers). The reasons for discontinuing the training were the following: a minor hip injury, an ankle tendon injury, a knee injury, illness unrelated to training, foot injury unrelated to training, skin disease unrelated to training, and family issue.

**Fig. 2:**
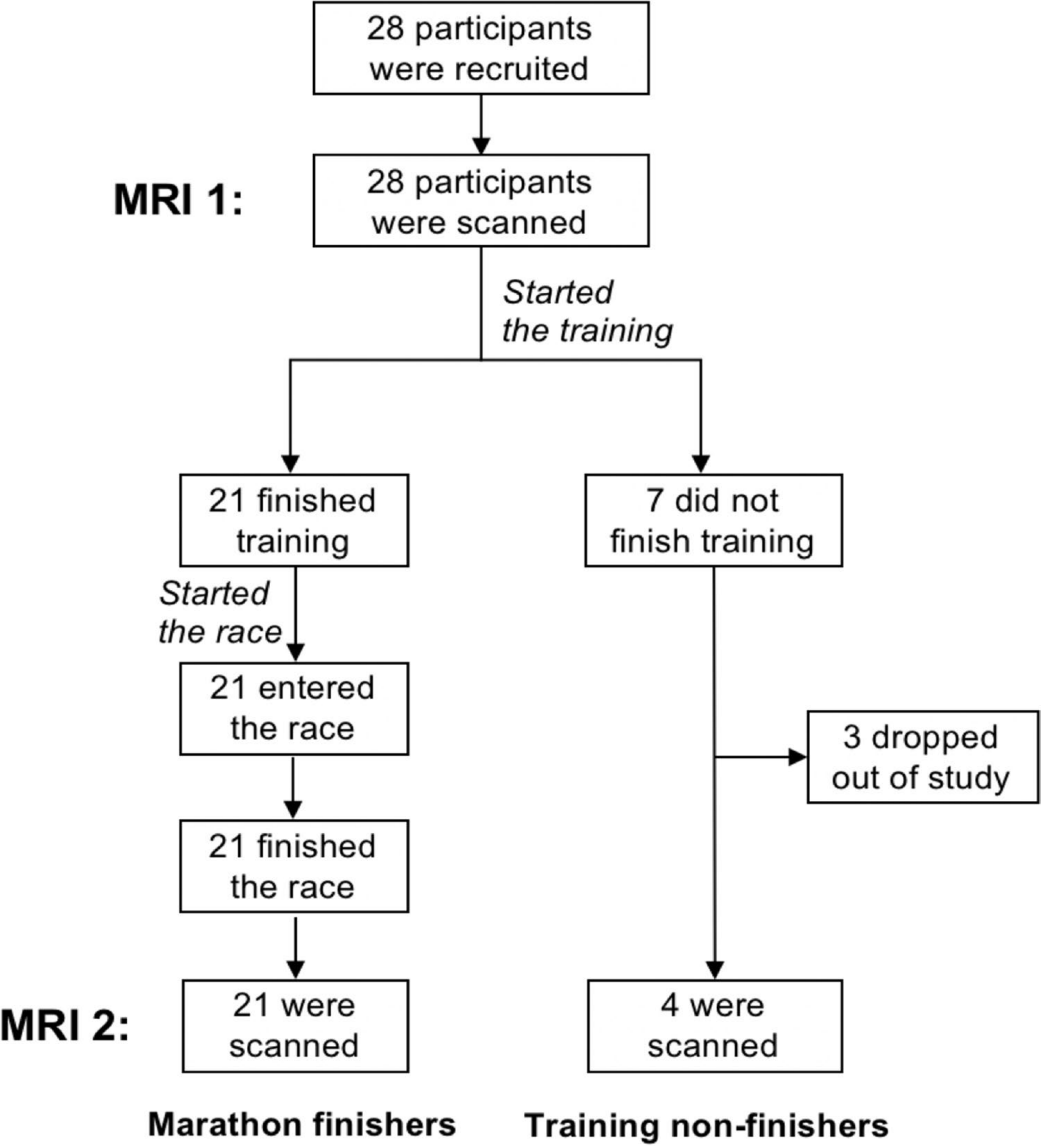
Study design

Following the race, all marathon finishers and 4/7 training non-finishers underwent another MRI scan. The 3 remaining training non-finishers did not return for MRI scanning because of personal issues of unavailability (see Fig. 2 for study design; further details in Appendix Table A1 and Table A2).

The mean of all marathon finishing times was 4 h 23 min ± 42 min. The presence of pre-training disc degeneration at time point 1 did not affect marathon finishing times when the finishing times of participants with disc degeneration and of those without disc degeneration were compared (*p* = 0.384).

### Time point 1 versus time point 2 changes

Measurements of lumbar spine features on MRI were taken at both time point 1 and time point 2: IVD height, IVD width, and intervertebral distance. These sets of data were compared and no significant changes were found from time point 1 to time point 2 i.e. changes were very small (see Table 3).

**Table 3:** Lumbar spine MRI changes between time point 1 and time point 2 in marathon finishers (*n* = 21) and training non-finishers (*n* = 4)

Parameters*	Marathon finishers (*n* = 21)			Training non-finishers (*n* = 4)		
	Time point 1	Time point 2	Change	Time point 1	Time point 2	Change
IVD height, mm	10.46	10.41	0.05	10.78	10.78	0
L3–L4	10.86	10.66	0.20	11.1	11.1	0
L4–L5	11.26	11.17	0.09	11.9	11.9	0
L5–S1	9.56	9.60	0.04	9.4	9.4	0
*p*-value	0.234			No difference		
IVD width, mm	33.09	32.97	0.12	30.41	30.41	0
L3–L4**	33.61	33.43	0.18	32.5	32.5	0
L4–L5	33.67	33.51	0.16	32.2	32.2	0
L5–S1	32.64	32.59	0.05	28.0	28.0	0
*p*-value	0.359			No difference		
Intervertebral distance, mm	24.93	24.93	0	26.13	26.13	0
L3	ns	ns	ns	ns	ns	0
L4	26.98	26.86	0.12	30.0	30.0	0
L5	24.63	24.54	0.09	25.6	25.6	0
S1	24.04	24.17	0.13	23.8	23.8	0
*p*-value	0.641			No difference		
Pfirrmann, grade (1–5)	1.66	1.66	0	1.71	1.71	0
L3–L4	1.46	1.46	0	1.66	1.66	0
L4–L5	1.62	1.62	0	1.75	1.75	0
L5–S1	1.86	1.86	0	1.75	1.75	0
*p*-value	No difference			No difference		
Facet joint effusion	2	2	0	0	0	0
Insufficiency fracture	0	0	0	0	0	0

Mean values of all measurements were calculated for each parameter.

*On 9 scans L3–L4 was not visible or was incompletely captured, whilst all the rest of participants’ scans fully captured L3–L4; therefore those specific 9 cases were not counted when the mean values were calculated as part of the analysis and were presented in the table.

**Fig. 3:**
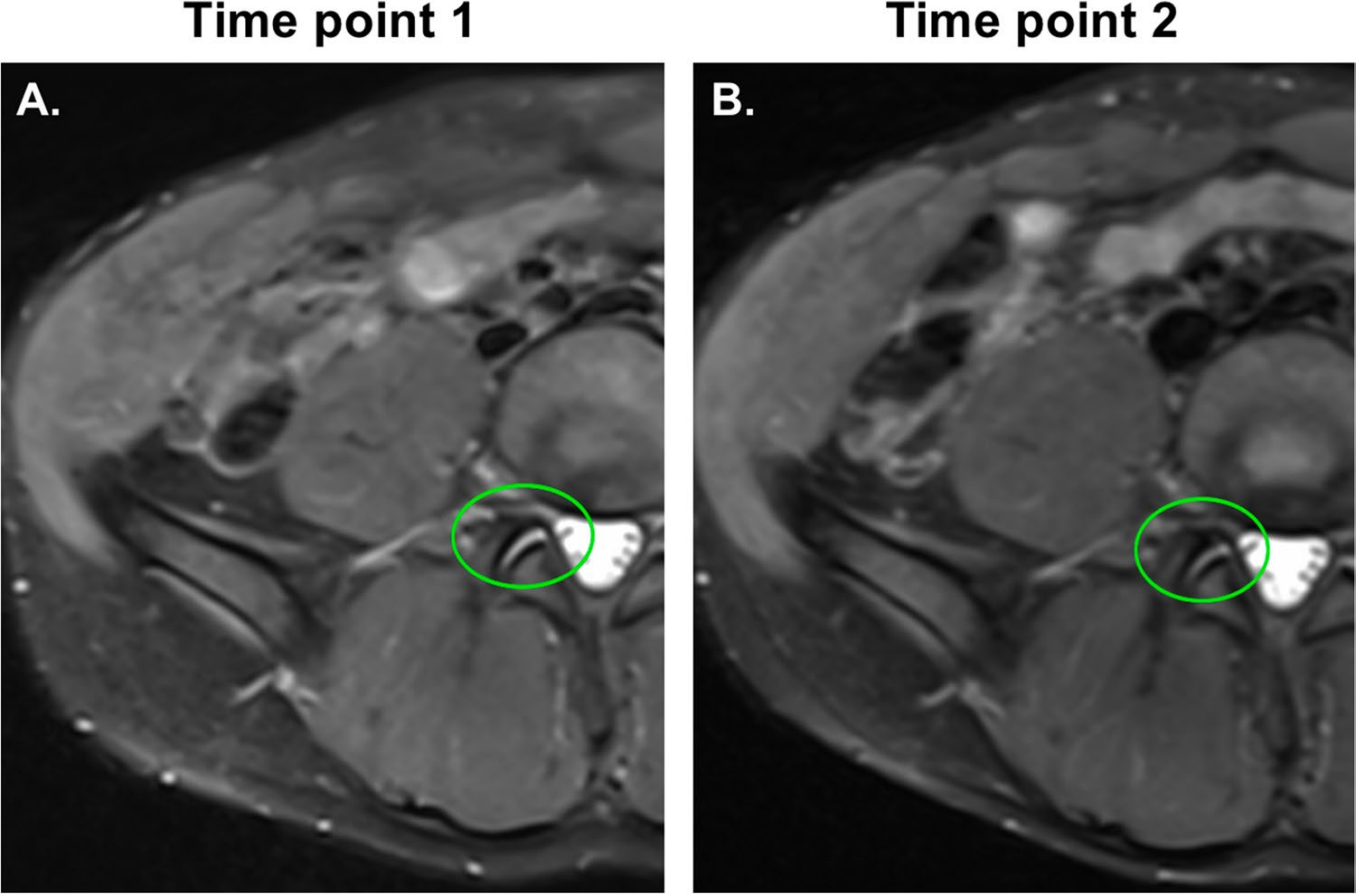
Axial Dixon PD FS TSE MR images of a 35-year-old man showing facet joint effusion at L4–L5 at time point 1 (**A**) and no worsening at time point 2 (**B**). No insufficiency fracture at either time point

Pfirrmann grades were unchanged between the two-time points. The two cases of facet joint effusion from time point 1 did not progress at time point 2 (Fig. 3). No new findings appeared at time point 2.

No significant differences were found between the grades/measurements reported for marathon finishers and those reported for training non-finishers (*p* = 0.690).

### Other incidental findings at the sacroiliac joint

At time point 1, there were a number of asymptomatic incidental findings in the sacroiliac joint: 8 cases of subchondral bone marrow oedema (4 in the iliac side, 4 in the sacrum), 2 cysts (one in the pubic symphysis and one in the sacrum), 2 cases of subchondral sclerosis and one sclerotic lesion in the iliac side, and 2 herniation pits.

**Fig. 4:**
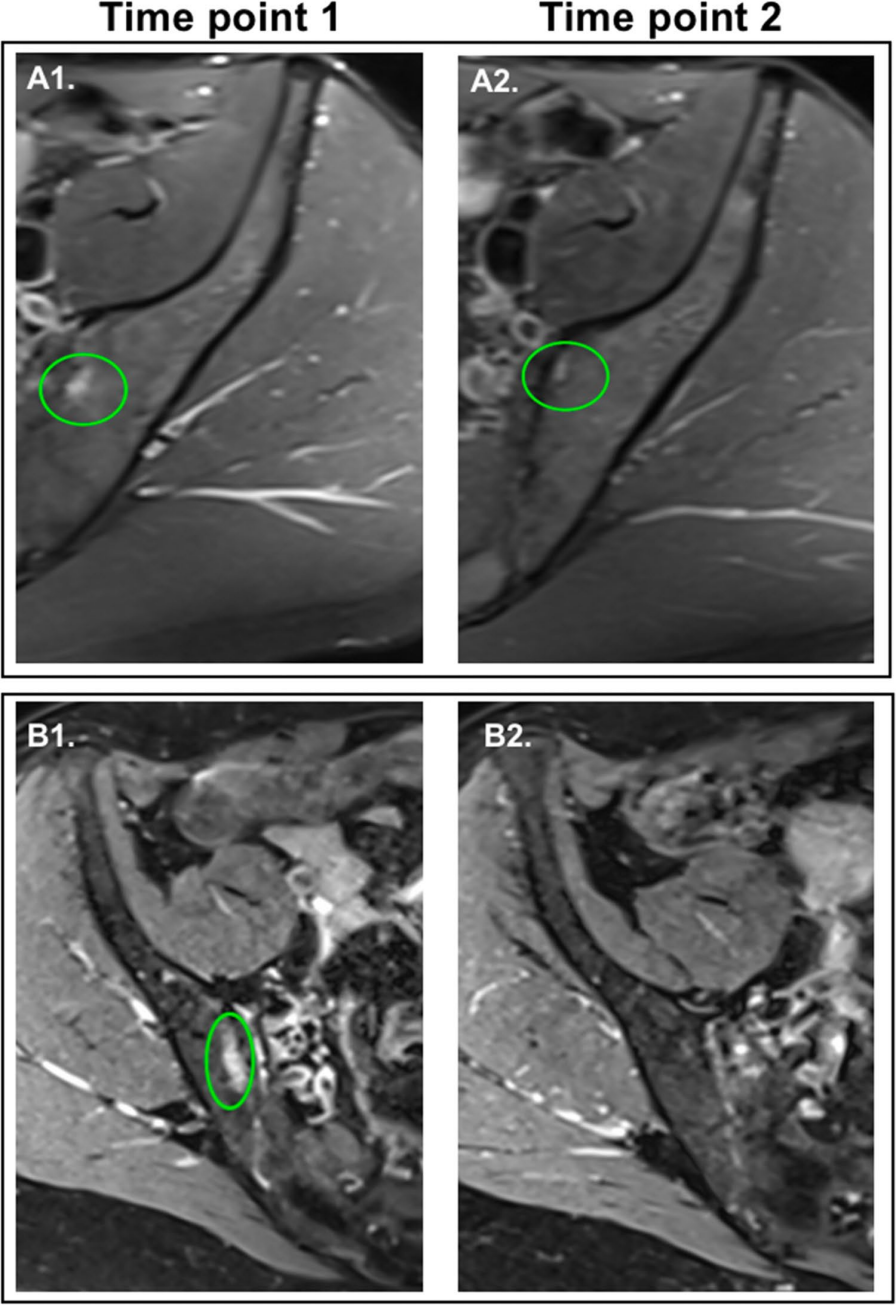
Axial Dixon PD FS TSE MR images of 2 participants: **A** one showing subchondral oedema in the iliac side of left sacroiliac joint at time point 1 (A1) which got smaller in size at time point 2 (A2) in a 35-year-old man; **B** one with oedema in the iliac side at time point 1 (B1) which disappears at time point 2 (B2) in a 58-year-old woman

At time point 2, most findings were unchanged on MRI apart from a few exceptions: one case of asymptomatic subchondral cyst in the sacrum slightly extended in size in comparison to time point 1, whilst 2/8 (25%) cases of bone marrow oedema showed regression, both in the iliac side, one showed a decrease in size, and the other one disappeared completely (Fig. 4).

There was perfect agreement between the 2 radiologists’ scores and measurements (kappa = 0.100). There were no particular findings giving rise to discordances.

### Participants characteristics

There were no significant differences between participants demographics and their corresponding disc condition (Pfirrman’s grades), in terms of: gender (*p* > 0.99), age (*p* = 0.21), and BMI (*p* = 0.91). The distinction was made between participants aged < 40 years old and those aged ≥ 40 years old, as well as between participants with BMI values of < 25 kg/m^2^ and ≥ 25 kg/m^2^, respectively.

The reduction of IVD height and IVD width, respectively, after marathon running was very little (see Table 4). The only significant difference was found for IVD width reduction in women, which was significantly higher than in men (*p* = 0.0007).

**Table 4:** Post-marathon IVD and BMI changes and corresponding participant demographics

Demographics	Marathon finishers, *n* (%)	IVD height reduction	IVD width reduction	BMI changes
Gender				
Males	12 (57%)	0.01	0.00	( +) 0.01
Females	9 (43%)	0.09	0.15	( −) 0.18
*p*-value	n/a	0.61	0.0007*	0.16
Age, years				
< 40	16	0.1	0.12	( −) 0.06
≥ 40	5	0.04	0.11	( −) 0.48
*p*-value	n/a	0.25	0.46	0.04*
BMI, kg/m^2^				
< 25	15	0.05	0.12	( −) 0.24
≥ 25	6	0.04	0.10	( −) 0.35
*p*-value	n/a	0.90	> 0.99	0.09

*Significant differences between different categories: *n/a*, not applicable; + , increase; − reduction; *BMI*, body mass index; *IVD*, intervertebral disc.

BMI changes occurred after marathon running, corresponding to weight reductions. There were no body height reductions. Significant BMI changes were only seen between different age groups (*p* = 0.04), and no associations with other demographics could be made (Table 4).

Regarding training non-finishers, no changes in IVD parameters or BMI were reported; therefore, no associations with demographics or other participant characteristics could be made.

No complaints of pain or others symptoms or functional limitations were reported by participants.

## Discussion

### Principal findings

A 3.0 T MRI of 21 healthy participants showed that running 500 miles over 4 months and then a marathon for the first time had no adverse effect on the lower lumbar spine, even when early pain-free degenerative changes were present. Additionally, sacroiliac bone marrow oedema (non-specific finding) was found in 8 participants at the start of the study and in 2 of them, this had regressed by the end. No participants dropped out of the study as a result of lumbar spine symptoms.

Before the start of training, 17/28 (61%) participants had spinal disc degeneration on MRI, especially at L4–L5 and L5–S1, although they were completely asymptomatic and had no back pain. Also, there were incidental findings in the sacroiliac joint including 8 cases of subchondral bone marrow oedema, 2 cysts, 2 subchondral sclerosis and one sclerotic lesion, 2 herniation pits, as well as 2 facet joint effusions. After the marathon, there were no changes in the spinal disc condition of runners on MRI. Also, there were no significant differences in the measurements of lumbar spine features (IVD height, IVD width, and intervertebral distance) between time point 1 and time point 2, but there was significant regression in the extent of sacroiliac joint bone marrow oedema in 2/8 (25%) cases at time point 2 whilst one pre-existing subchondral cyst slightly increased in size, yet this was a minor change. No symptoms of low back pain or other complaints were reported by participants.

### Comparison with previous studies

Research on running-related effects on the lumbar spine is very limited. Moreover, no previous study investigated the impact of marathon running on the lumbar spine before and after the marathon. Therefore, no direct comparisons can be made.

In accordance with our study, in a cross-sectional pilot study, no negative radiological findings were seen in the lumbar spines of middle-aged long-term endurance runners [3]. In fact, improved IVD appearances were detected in runners with an increased number of years of running experience and weekly running distances. Other running studies also showed the beneficial impact of running on IVD health in participants who had daily moderate to vigorous-intensity running activity [10], as well the regular weekly running activity of 20–40 km/week and > 50 km/week [9].

Very few studies showed opposite findings. One study showed that there was a reduction in IVD height and volume in young adults after 30 min of moderate-intensity running; nevertheless, the sample size was small (*n* = 8) and various confounding factors may need to be considered [11]. Also, a retrospective review of patient data suggested that Olympic Games athletes had a high number of degenerative disc diseases of cervical and lumbar spines on their MRIs. However, the athletes were symptomatic and did not include specifically runners [12].

### Strengths and weaknesses

To our knowledge, this is the only study that has assessed the impact of marathon running on the lumbar spine of runners. There are several study strengths: 1)The study sample included first-time marathon runners who undertook a 4-month training programme to minimise the additional effects of pre-study running experience; 2) The equipment used was high-resolution 3.0 T MRI and 2 musculoskeletal radiologists reported the findings, with perfect agreement (kappa = 0.100) following validated scoring systems; 3) Detailed analysis of lumbar spine features was done, providing a comprehensive evaluation of the health status of the spine both before and after the marathon run.

The following study limitations were acknowledged: 1) radiological evaluation cannot exclude a certain degree of bias. To reduce the likelihood of this happening, 2 radiologists were involved in the study to report the MRI findings and do the IVD measurements: one radiologist evaluated all images, then a subset of images were double-reported by a 2nd radiologist, independently, and there was a perfect interreader agreement; 2) 3 training non-finishers were not available to attend the time point 2 MRI scan, therefore comparisons between pre- and post-training datasets could not be made. Nevertheless, only one of these had a knee issue during training which led to training cessation, whilst the rest did not discontinue the training for running-related reasons; 4) MRI scanning did not capture L1, L2 nor L1–L2 and L2–L3 discs; additionally L3–L4 was not scanned (or was incompletely scanned) in 9 cases. This did not allow for a full analysis of the lumbar spine. However, data from literature suggests that the segments most commonly vulnerable to degeneration are L4–L5 and L5–S1, due to compression forces [13–15] which was confirmed in this study as well (and were fully scanned); 5) Disc bulging or nerve compression could not be evaluated based on the MRI protocol used (there were no dedicated slices through the discs); also, disc volume and vertebral height were not evaluated as part of the study; 6) a long-term follow-up is required to monitor changes in IVD measurements and disc condition over time, as well as any changes in symptoms reported by participants; 7) only post-race MRI scanning was done, but no immediate post-training scanning (right before the race); this could have revealed the impact of training alone on the spine, however the aim of the study was to evaluate the findings after the marathon training plus race altogether; 8) The clinical significance of lumbar disc degeneration seen on time point 1 MRI scans of asymptomatic individuals (before training) remains unclear. The findings may help in understanding how to better interpret the importance of degenerative radiological findings and may support clinical decision-making, however, long-term monitoring is required to be able to draw better conclusions.

## Conclusion

A 3.0 T MRI showed that running 500 miles over 4 months and then a marathon for the first time had no adverse effect on the lower lumbar spine, even when early symptom-free degenerative changes were present. Additionally, there was some evidence that few abnormalities of the sacroiliac joints regress.

## Supplementary Information

Below is the link to the electronic supplementary material.

Supplementary file1 (DOCX 13 KB)
