# Supplementary material for: What happens to the lower lumbar spine after marathon running: a 3.0 T MRI study of 21 first-time marathoners
Source: Skeletal Radiol. 2021 Sep 20;51(5):971–80. doi: 10.1007/s00256-021-03906-5 (PMC8930792; doi:10.1007/s00256-021-03906-5)
Supplement: Supplementary file 1 — Supplementary file1 (DOCX 13 KB) [file 256_2021_3906_MOESM1_ESM.docx]

**Table A1: Additional information on training non-finishers who attended both time point 1 and time point 2 MRIs (n=4)**

| **No. (participant)** | **Reasons for discontinuing**  **training** | **Number of months of training completed before discontinuing** | **Training-related symptoms resolved**  **by time point 2** |  |
| --- | --- | --- | --- | --- |
| 1  2  3  4 | Hip injury related to training | 2 | Yes |  |
|  | Ankle injury related to training | 2 | Yes |  |
|  | Illness unrelated to training | 3 | Not applicable |  |
|  | Foot injury unrelated to training | 2.5 | Not applicable |  |

**Table A2: Additional information on training non-finishers who attended time point 1 MRI only, but not time point 2 MRI (n=3)**

| **No. (participant)** | **Reasons for discontinuing**  **training** | **Number of months of training completed before discontinuing** | **Training-related symptoms resolved following training discontinuation** |  |
| --- | --- | --- | --- | --- |
| 1 | Knee injury related to training | 2 | Yes |  |
| 2 | Skin disease unrelated to training | 3.5 | Not applicable |  |
| 3 | Family issue | 3 | Not applicable |  |
